# Southeast Asia after a year: what lies ahead for the health of the region?

**DOI:** 10.1016/j.lansea.2023.100243

**Published:** 2023-06-12

**Authors:** The Lancet Regional Health – Southeast Asia

As part of the global health equity initiative of the Lancet Group, *The Lancet Regional Health — Southeast Asia* was launched as a platform to provide voice to local researchers, clinicians, policy makers, and most importantly patients and general readers. The inaugural Editorial committed to advocate for building a better, fairer, and healthier future for the nearly 2 billion people in the WHO South-East Asia region.

The past year was marked with significant social, political, and economic events as life started getting back to normal after 3 years of the COVID-19 pandemic. India overtook China to become the most populous country in the world. India also took over the G20 presidency from Indonesia at time of geopolitical turmoil and uncertainty over post-pandemic economic recovery. Economic and political crisis in Sri Lanka led to shortages of medical supplies, and an increase in prices of basic commodities. Thailand became the first country in Southeast Asia to legalise marijuana for medical purposes. Extreme climate events such as heatwaves, cyclones, and floods devastated and displaced millions. Sporadic outbreaks of cholera, mpox, dengue, Ebola, and Marburg viruses in various parts of the world continued to keep the governments and public health fraternity on their toes. Early last month, WHO declared that COVID-19 and mpox are no longer public health emergencies of international concern.

Several high-quality pieces highlighted the pandemic's lasting impact on care and services, particularly routine immunisation and education. Randomised trials and large cohort studies on COVID-19 demonstrated the severity and mortality of the disease, evaluated treatments and various therapeutics, and assessed vaccine safety and efficacy in populations of almost all countries of the region, including Timor-Leste and Bhutan. Research attempted to estimate the severity and mortality associated with COVID-19 among children in India and Pakistan. As the focus started to shift away from COVID-19 vaccines and treatments, researchers are now working on topics such as wastewater surveillance, pandemic preparedness, vaccine production technology, and policy response in case of future outbreaks.

WHO designated 2021 as the International Year of Health and Care Workers to appreciate their sacrifice and services in the difficult times of COVID-19. Our August Editorial saluted the importance of a workforce who continued to endure several physical health problems, mental health problems, and violence, among many other challenges during the pandemic. WHO also recognised ASHAs (frontline workers in India) as Global Health Leaders at the 75th World Health Assembly last year. A strong Comment tried to unpack the meaning of this recognition and reflect on the relative power and agency of ASHAs in the hierarchical and power-laden health systems in India.

Mental health is one of the topics that the journal will continue to prioritise over the next few years. Innovative models such as a transdisciplinary intervention in the community are being tested among children and adolescents who had experienced sexual and physical abuse. Another study in Myanmar assessed an intervention aimed at upskilling and empowering community health workers to screen and diagnose mental health issues in the community. A striking piece on harm reduction policy of opium-rich Afghanistan talks about government action even during times of political and economic turmoil. The only country in the world with a Gross National Happiness Index as their growth indicator, Bhutan, also reported a high burden of depression during the pandemic. Researchers recommend short-term and long-term measures to cater to the growing impact of ongoing political and economic turmoil on the mental health of children and youth in Sri Lanka. A correspondence from Pakistan critiqued the universal health coverage (UHC) policy for excluding the population with mental health issues, missing an opportunity to put mental health financing on the map of social security.

The journal pledged to address inequity not only in research but also in publishing and wider society. The journal resolved to publish articles written mostly by local researchers, reviewed by local research community, and most importantly curated by local editors. The journal demonstrated its commitment to advocate for health rights of diverse populations by publishing good quality research on disadvantaged communities: highlighting the needs of non-binary people who menstruate, LGBTQ+ people, transgender groups, and the girl child. Several research articles and opinion pieces strived to bring out the discrimination of migrants and refugees, particularly Rohingyas who face a human rights crisis, as well as child protection issues and mental health issues in Bangladesh. Our November Editorial advocated strongly for governments to strengthen legislation and support systems to eliminate violence in any form against women. Child marriages, for example, demonstrate that the role of inequitable gender norms and their impact on mental health and suicide risks among girls and young women in the region is enormous.

In this first anniversary issue, an encouraging piece on evolving mental health research by Dr Lakshmi Vijaykumar laments the highest prevalent suicide rates in India and the need for more community engagement to prevent, treat, and rehabilitate mental health issues.

As we look forward to moving to integrating digital innovations into health care and research, Dr Anurag Agarwal discusses the challenges and opportunities of generative AI and its foundations, and how its widespread use in routine health care and research would uphold the principles of accountability, transparency, safety, equity, and sustainability.

As we move out of the pandemic, it is clear that we need accelerated policy response to provide UHC with efficient allocation and better management. Developing a strategic roadmap at this time will help to ensure success with regard to meeting the SDGs. The journal will continue its commitment to global equity by providing an open access platform for publishing high-quality evidence that helps clinicians, patients, and policy makers from the region make informed decisions for a better, stronger, and healthier future. The journal will also continue to promote regional collaboration and collective learning and engagement for dealing with current health and future emergencies.

